# Thermal Analysis of Blood Flow Alterations in Human Hand and Foot Based on Vascular-Porous Media Model

**DOI:** 10.3389/fbioe.2021.786615

**Published:** 2022-01-28

**Authors:** Yue-Ping Wang, Rui-Hao Cheng, Ying He, Li-Zhong Mu

**Affiliations:** School of Energy and Power Engineering, Dalian University of Technology, Dalian, China

**Keywords:** thermal analysis, vascular disorder, blood flow estimation, diabetic foot, porous media model

## Abstract

Microvascular and Macrovascular diseases are serious complications of diabetic mellitus, which significantly affect the life quality of diabetic patients. Quantitative description of the relationship between temperature and blood flow is considerably important for non-invasive detection of blood vessel structural and functional lesions. In this study, thermal analysis has been employed to predict blood flow alterations in a foot and a cubic skin model successively by using a discrete vessel-porous media model and further compared the blood flows in 31 diabetic patients. The tissue is regarded as porous media whose liquid phase represents the blood flow in capillaries and solid phase refers to the tissue part. Discrete vascular segments composed of arteries, arterioles, veins, and venules were embedded in the foot model. In the foot thermal analysis, the temperature distributions with different inlet vascular stenosis were simulated. The local temperature area sensitive to the reduction of perfusion was obtained under different inlet blood flow conditions. The discrete vascular-porous media model was further applied in the assessment of the skin blood flow by coupling the measured skin temperatures of diabetic patients and an inverse method. In comparison with the estimated blood flows among the diabetic patients, delayed blood flow regulation was found in some of diabetic patients, implying that there may be some vascular disorders in these patients. The conclusion confirms the one in our previous experiment on diabetic rats. Most of the patients predicted to be with vascular disorders were diagnosed as vascular complication in clinical settings as well, suggesting the potential applications of the vascular-porous media model in health management of diabetic patients.

## Introduction

Due to lifestyle changes, reduced physical activity, and increased obesity, the prevalence of diabetes has increased from 4.7% in 1980 to 8.5% in 2014. It is estimated that there will be more than 629 million adult diabetic patients in 2045 (Glovaci et al., 2019). Diabetic foot is one of the common and dangerous complications of diabetes mellitus, with an incidence rate of 6.3% worldwide (Zhang et al., 2017). All layers of tissues from the skin to bones will be affected by ulcers. In severe cases, amputations are required and even contralateral foot wound or repeated amputations may be induced, which not only reduces the life qualities of patients but also causes huge medical pressure and economic losses. Therefore, early detection of diabetic foot is of vital importance. Diabetic complications are always accompanied with structural and functional disorders of the peripheral vascular system. Orchard and Strandness, (1993) found that calcification occurs in the posterior tibial artery, anterior tibial artery, and the arteries at the plantar level among diabetic patients through X-ray. The atherosclerotic plaque leads to occlusion of blood vessels. The vascular occlusion may reduce blood flow and further obstruct the transport of active substances which induce the onset of foot ulceration. Additionally, abnormal hemodynamic and metabolic dysfunctions contribute to the autoregulation of vasomotion disorders and eventually result in ischemia which would intensify ulceration. Therefore, the key factor of early diagnosis of diabetic foot is to detect the dysfunctions of macro/microvasculature as early as possible.

The methods for clinical evaluation of the lower extremity arterial disease include intermittent claudication observation, foot arterial pulsation measurement, ankle-brachial blood pressure index measurement, and so on. Among them, the ankle-brachial index (ankle-brachial index, ABI) is a proven reproducible inspection method. A hand-held Doppler probe can be used to measure the systolic blood pressure of the ankle and arms, and then calculate the ratio of the two. This operation is simple and non-invasive, and the results have been verified in the lesions confirmed by angiography. However, in some elderly patients, calcium deposition in the middle arteries and poor vascular compressibility may occur, resulting in an increase in the ABI, leading to false normals. The mainstream non-invasive methods for detecting the microcirculation blood perfusion rate include the Doppler effect–based laser Doppler flowmetry (laser Doppler flowmetry, LDF) direct detection and the transcutaneous oxygen content that indirectly reflects the perfusion rate through the partial pressure of oxygen. The high price of the abovementioned instruments and poor portability limit their wide application. On the other hand, plethysmograph (Schürmann et al., 2001), laser speckle imager (Briers, 1996), and other blood perfusion rate detection methods based on mechanical and ultrasound technology are still immature. Therefore, effective methods for non-invasive detection of vascular disease in diabetic patients still need further study.

Skin temperature variation is closely associated with the blood perfusion rate, suggesting that monitoring temperature alterations may be employed to study vascular reactivity. Through wavelet cross-correlation analysis of laser Doppler flowmetry (LDF) and skin temperature signal in healthy subjects, Frick et al. pointed out skin temperature monitoring can be used as a tracer of microvessel tone (Frick et al., 2015). The response to the cold pressor test in patients with type 2 diabetes differs essentially from that of healthy subjects in the endothelial frequency range (Smirnova et al., 2013). Podtaev et al. analyzed the correlation degree and phase shift between skin temperature fluctuations and periodic changes of the blood flow caused by oscillations in vasomotor smooth muscle tone (Podtaev et al., 2008). Thermography can be conveniently transformed into skin blood flow through a new developed spectral filter approach (Sagaidachnyi et al., 2017). Nieuwenhoff et al. (2016) also found the time constant expressing skin temperature variation rate can reflect the blood flow of the skin through the skin temperature heating test and heat transfer modeling. Reproducibility was confirmed in the assessment of axon reflex-related vasodilation. Coupling with thermography of tongue and bioheat transfer analysis, the state of the lingual circulation system can be assessed, which provides evidence for the traditional diagnosis method *via* observing the tongue surface state in Chinese medicine (Zhang and Zhu, 2010).

In the past 2 decades, various medical instruments for detecting pathological conditions in the circulatory system have been developed based on the correlation between temperature and blood flow, some of which are listed in Table 1 showing the devices, experimental thermal environment, experimental subjects, and analytical models. Haga et al. (2012) developed an instrument and algorithm for estimation of blood perfusion from the measured skin temperature. A similar fingertip temperature measurement instrument has been also designed, which can record the temperature change within 75 s after the fingertip touches the sensor (Nagata et al., 2009). The developed heat transfer model was in analogy with the circuit where the thermal conductivity corresponds to the electrical resistance. As blood flow of capillaries affect the effective thermal conductivity of the skin, blood perfusion could be further inferred from the resistance value in the circuit model. In Wang et al.’s heating experiments on diabetic rats implemented by a microtest device, the blood perfusion before and after heating was evaluated by coupling a 1D bioheat transfer model and genetic algorithm (Wang et al., 2020). Apart from the conventional genetic algorithm, the Box–Kanemasu method (Ricketts et al., 2008) was also employed for prediction of blood perfusion from measured temperatures in Rickettes et al.’s study. It is seen a common feature from the abovementioned studies that various optimization algorithms have been used to estimate the blood perfusion rate or thermophysical parameters based on the surface temperature. This approach can be categorized as inverse analysis which is distinguished from the forward one to compute the surface and depth temperature distributions by using the given boundary conditions. In order to estimate these parameters, choosing a suitable heat transfer model for matching the added surface heating source is a key factor as well.

**TABLE 1:** Experimental study on the thermal method of vasomotor function.

Experimental device	Thermal environment	Experimental subject	Analytical method	References
Holdable heat-stimulated blood flow test instrument	Temperature measurement with local heating and recovery	Healthy people’s hand	2-D cylindrical tissue model in a cylindrical coordinate system	Haga et al. (2012)
Fingertip temperature dual sensor	No external thermal stimulation	Healthy people’s finger tip	0-D parametric model analogous to a circuit	Nagata et al. (2009)
Microtest	Temperature measurement with local heating and recovery	Healthy and diabetic SD rats’ paw	1-D vascular-porous media bioheat transfer model	Wang et al. (2020)
A laminated flat thermocouple sensor	No external thermal stimulation	Healthy rats’ liver tissue	2-D finite difference tissue heat transfer model	Ricketts et al. (2008)
14-node thermal mapping sensors	Temperature measurement with local heating and recovery	Healthy people’s arm	2-D finite element bioheat transfer model	Webb et al. (2015)
Coupling of the optical probe with the Peltier element	Temperature measurement with local heating and recovery	Healthy and diabetic people’s lower limb	Spectral analysis by using a wavelet transform	Mizeva et al. (2018)
Infrared thermography and photoplethysmography	No external thermal stimulation	Healthy people’s fingertip	Morelet wavelet transfrom	Sagaidachnyi et al. (2014)
Temperature sensorHRTS-5760, Honeywell International, Inc., United States	Temperature measurement with local heating and recovery	Healthy and diabetic people’s palm	Wavelet analysis of temperature	Podtaev et al. (2014)
Microtest	Temperature measurement with local heating and recovery	Healthy people’s/diabetic patients’ finger tip	Wavelet analysis of temperature	Zubareva et al. (2019), Parshakov et al. (2016), Parshakov et al. (2017), Antonova et al. (2016)

With the development of thermal imaging technology, a lot of research practices have been carried out around computer-aid diagnosis of diabetic foot by infrared thermography (Hernandez-Contreras et al., 2016; Muhammad et al., 2017). Comparing with other imaging modalities such as MRI, CT, and ultrasound, infrared thermal imaging is safer and more convenient (Bandalakunta Gururajarao et al., 2019). In the circumstance of critical ischemia, as a non-invasive method, thermal imaging is more effective than the toe brachial pressure index (Kevin et al., 2019). Sivanandam et al. (2012) collected the infrared thermal image of plantar among healthy people and diabetic patients with and without early signs of ulceration. It is found that the foot temperature of a diabetic subject without the complication of ulceration was 2°C lower than that of the healthy subjects. The average foot skin temperature in diabetic patients with early signs of ulceration decreased by 0.5°C when compared with control subjects. The results suggest that diabetic patients with vascular complications and early signs of ulceration present different variation mechanisms in temperature distribution, of which one is mainly due to blood flow rate decreasing, but another may be caused by the occurrence of inflammation. Bagavathiappan et al. (2008) observed temperature gradients in the influenced regions of patients with vascular disorders and ischemic gangrene from thermal imaging. It was also displayed that diabetic subjects with neuropathy had higher mean foot temperature than non-neuropathic subjects (Bagavathiappan et al., 2010). The thermal imaging analyses show that there is usually a rapid rise about 0.7°C in skin temperature when a local wound occurs. If an inflammation occurs, the skin temperature will increase by 2.2°C (Chen et al., 2021). Van Netten et al. (2013) explored the applicability of infrared thermal imaging for detection of signs of diabetic foot by comparing the mean temperature between the lateral and the contralateral foot and found that the mean temperature difference of the feet in diabetic patients with diffusive complications is larger than 3°C.

Astasio et al. obtained thermograms of the sole in 277 diabetics and analyzed the temperature distribution patterns in four areas of the soles (Astasio et al., 2018) Additionally, they found a much lower mean temperature of soles in diabetics by further comparisons of the thermal maps with those of nondiabetics (Astasio-Picado et al., 2020). Using combined discrete wavelet transform and higher order spectra techniques (Muhammad et al., 2018a) or double density-dual tree-complex wavelet transform (Muhammad et al., 2018b), original foot thermal images can be decomposed for providing various valuable information in the diagnosis of the diabetic foot. The application of machine learning into infrared image processing can improve the accuracy and speed for classification of diabetic foot thermograms (He et al., 2021). In the future, a neural network model can be inserted into mobile phones for early detection of diabetic ulcers after training by using numerous foot temperature data for diabetic ulcer patients and healthy subjects (Serlina et al., 2020) (Amith et al., 2021). New techniques continuously appear to help identifying risk zones of diabetic foot, such as using a retrained MASK-R-CNN mode (Maldonado et al., 2020). In choosing the training data, it is pointed out that the temperatures of toes and the upper half of foot are better than those in other regions (Carlos Padierna et al., 2020) Although average skin temperature is a meaningful index for early diagnosis of diabetic foot in diabetic patients, it is rather insufficient to distinguish different stages of early signs. The foot temperature of diabetic patients with only vascular disease is frequently lower than that of healthy people. However, when it further develops into neuropathy, there will be an increase in the local foot temperature, which is associated with early inflammation in some places. Moreover, multiple vascular stenoses will have varying degrees of impact on each local foot temperature. Therefore, spatial variations of skin temperature should be more concerned. It is without doubt to see that the thermography-based diagnosis technique is powerful for detection of early stages of diabetic foot. However, few research studies concern with the underlying mechanisms associated with diabetic foot, such as the coherence between the altered foot vasculatures and tissue wound or the influence of arterial occlusion on blood perfusion in tissues. It has been known that skin temperature variations are closely associated with the variations of blood perfusion. Despite that detailed temperature changes can be detected using current thermography-based techniques, it is still difficult to define the serious degrees of the diseased vascular system simply based on thermal images.

In this regard, bio-heat transfer modeling is helpful for establishment of a quantitative relationship between blood flow rates and temperature distribution. This kind of work has been extensively explored in numerous available literature reports. Ma et al. (2015) simplified foot tissue as a three-layer structure of skin, fat, and a core zone and gave an analytical mathematical solution for the simplified one-dimensional case. In Copetti et al.’s study (Copetti et al., 2017), thermal analysis on the foot was carried out by using a two-dimensional finite element model. However, dimensionality reduction results in the loss of complete three-dimensional (3D) temperature information. Rafael et al. (2016) presented a 3D-finite element simulation to predict the temperature variations of the foot with 5 ulcers at the depth of 5 mm away from the sole of the foot. Although the remarkably higher temperatures in ulcers have been achieved, the influence of altered blood flow and vasculatures on tissue temperatures has not been taken into account. No matter the above 2D or 3D heat transfer computation, they were all performed by using a one Pennes equation, and the thermal effect of blood phase is reflected in the blood perfusion term (Pennes, 1998). Due to the simplicity of the Pennes equation, it has been widely used in macroscale bio-heat transfer computation such as hand (Shao et al., 2014) and even thermal characteristics of the whole body (Tang et al., 2016) which has clinical significance such as the treatment of breast tumor by using hyperthermia therapy (Barrios et al., 2019). However, determination of the local blood perfusion rate in the tissue is always a challenging work, especially in pathological conditions when the blood perfusion rate becomes non-uniform. Another limitation of the Pennes equation is that the influence of blood flow directions is neglected, resulting in over or under estimations of blood perfusion in some conditions. The discrete vascular bioheat transfer model provides an alternative method to compute the non-uniform local blood perfusion.

The important feature of the discrete vascular bioheat transfer model is to consider the vessels as a separate domain and calculate the heat exchange between the surrounding tissues. Among them, embedded 1D/3D multiscale modeling has been extensively employed to deal with heat transfer between vessels and tissues. By using this kind of model, Tang et al. (2020) computed the distribution of oxygen and temperature distribution in microcirculation. The influence of the red blood cell on oxygen transport can be further addressed in the work of Wang et al. (2021). He and Liu, (2017) proposed a coupled continuum-discrete model (CCD) for thermal analysis. They classified the blood vessels as visible vessels and invisible vessels. The thermal effect of visible vessels includes blood heat transfer and the conduction between the blood vessel and the surrounding tissue. The effect of invisible vessels was converted to the blood perfusion term corresponding to the continuum parts. The CCD model can well capture a richer and complex thermal interaction of the vascular network and solid tissue compared to the conventional bioheat transfer model. Stephen studied the effect of surface cooling on the internal temperature of the brain by inserting a one-dimensional vascular structure into the brain region (Blowers, 2018; Blowers et al., 2018). The 1D vascular model contains the arteries, arterioles, veins, and venules. The capillaries were represented by the liquid phase of porous media whose solid phase corresponds to the white or gray matter of the brain. It is found from their study that due to the inclusion of the directional flow, scalp cooling has a larger impact on cerebral temperatures than the predictions by previous bioheat transfer models. Although the discrete vascular-porous media model is more complex than the Pennes equation, it can describe the biological heat transfer process more realistically, and the inversion of the blood flow is more reliable. Image-based voxel mesh generation provides an easy-implemented way to apply the discrete vascular-porous media model in the analysis of the real geometric structure.

In this study, the discrete vascular-porous media bioheat transfer model has been applied in thermal analysis on a cubic tissue model and a foot to evaluate the influence of the blood flow with various vasculatures. The tissue is regarded as a porous media, while the embedded vasculature includes arteries, arterioles, venules, and veins. The conductive and convective effects of blood flow in multi-scaled blood vessels on tissue temperature are well-addressed in the model. The temperature distributions for various degrees of foot vascular stenosis were simulated, and the relationship between the foot temperature distribution and blood flow was quantitatively correlated. A cubic porous media model embedded with the vessel network was also coupled with the measured skin temperature data for analysis of blood flow regulation in diabetic patients. The blood flow in the conditions of skin heating and power off were estimated according to the test setting, and comparisons of the predicted blood flow were made between healthy people and diabetic patients.

## Methods

### The Developed Portable Thermal Sensor for Skin Temperature Measurement

**FIGURE 1:**
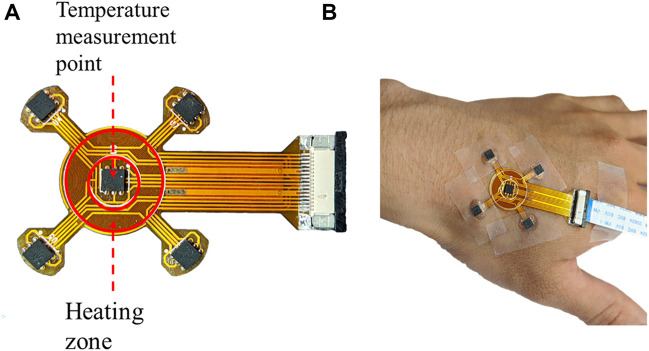
**(A)** Display of the SPAS sensor and **(B)** its temperature measurement process during fixed at a hand.

In order to compare the automatic regulation of the blood flow between diabetic patients and healthy people, a programmable heating and temperature measurement device using a flexible material has been developed which is named by the superficial perfusion assessment system (SPAS). As shown in Figure 1A, the substrate of the SPAS is made of a double-layer flexible printed circuit (FPC) board, which is formed by 3 layers of polyimide, 2 layers of copper foil, and 4 layers of adhesive bonding. The top layer is welded with a high-precision temperature sensor chip si7051 and FPC connector. The circuit that is coiled into a loop near the center of the bottom layer generates heat when energized, which thermally stimulates the surface of the skin. The detailed content about the production and debugging of the film can be obtained from Cheng’s master thesis (Cheng, 2020).

In the measurement, a medical tape was used to fix the SPAS on the skin of a hand, as shown in Figure 1B. Since the movement of a hand may cause the deviation of the flexible sensor, the subject should remain as stable as possible during the test. After manually turning on the device, the measurement will start and end automatically when the setting period is reached. The duration of an experiment is set to 2,750 s including 3 stages. In the first 750 s, the heating power is 0 W/m2, which is called the resting phase. Then, it comes to the heating phase by heating the skin with a power of 150 W/m^2^ for 1,000 s. After the power is switched off, the SPAS continues to record the temperature for another 1,000 s referring to the recovery phase.

### Geometric Models and Mathematical Descriptions of the Vascular Porous Media Model

**FIGURE 2:**
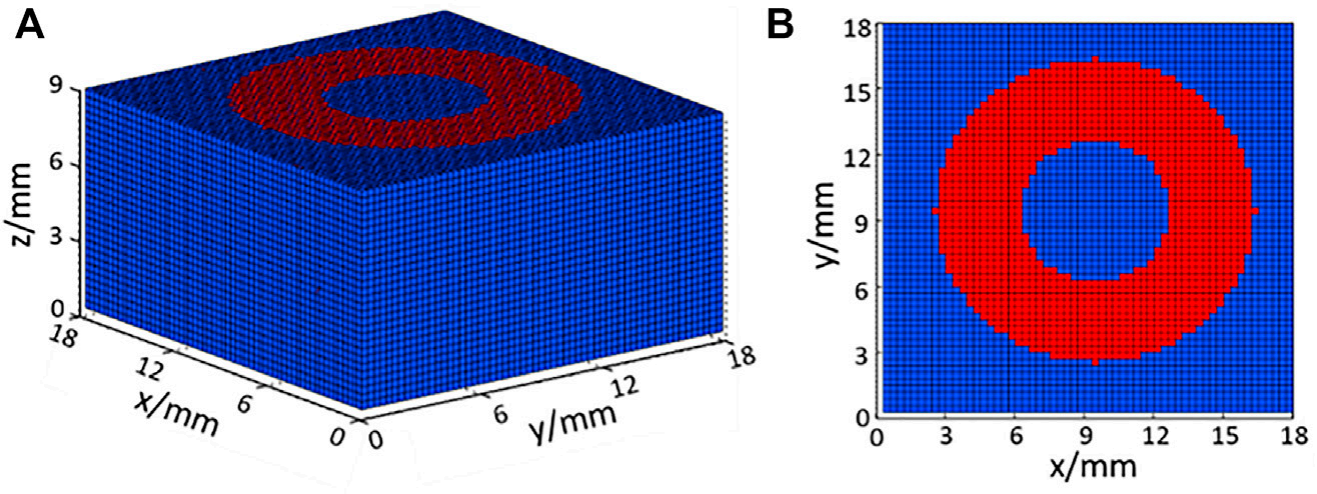
**(A)** Three-dimensional cubic tissue model with voxel meshes and **(B)** its bottom view.

According to the shape and size of the SPAS equipment, a cubic tissue model was designed, as shown in Figure 2A which could describe the heat exchange between the skin surface and deeper tissue. The volume of the tissue model is 1.8 × 1.8 × 0.9 cm^3^, and the voxel size is set as 0.3 mm. The upper surface of the model is the skin and conducts convective heat exchange with the surrounding environment. As shown in Figure 2B, the red area corresponds to the heating ring of the SPAS whose inner radius is 3.5 mm, and the outer one is 6.9 mm. An input heat flux as 150 W/m^2^ is imposed on the red region at the heating phase, and zero is set in resting and recovery phases.

**FIGURE 3:**
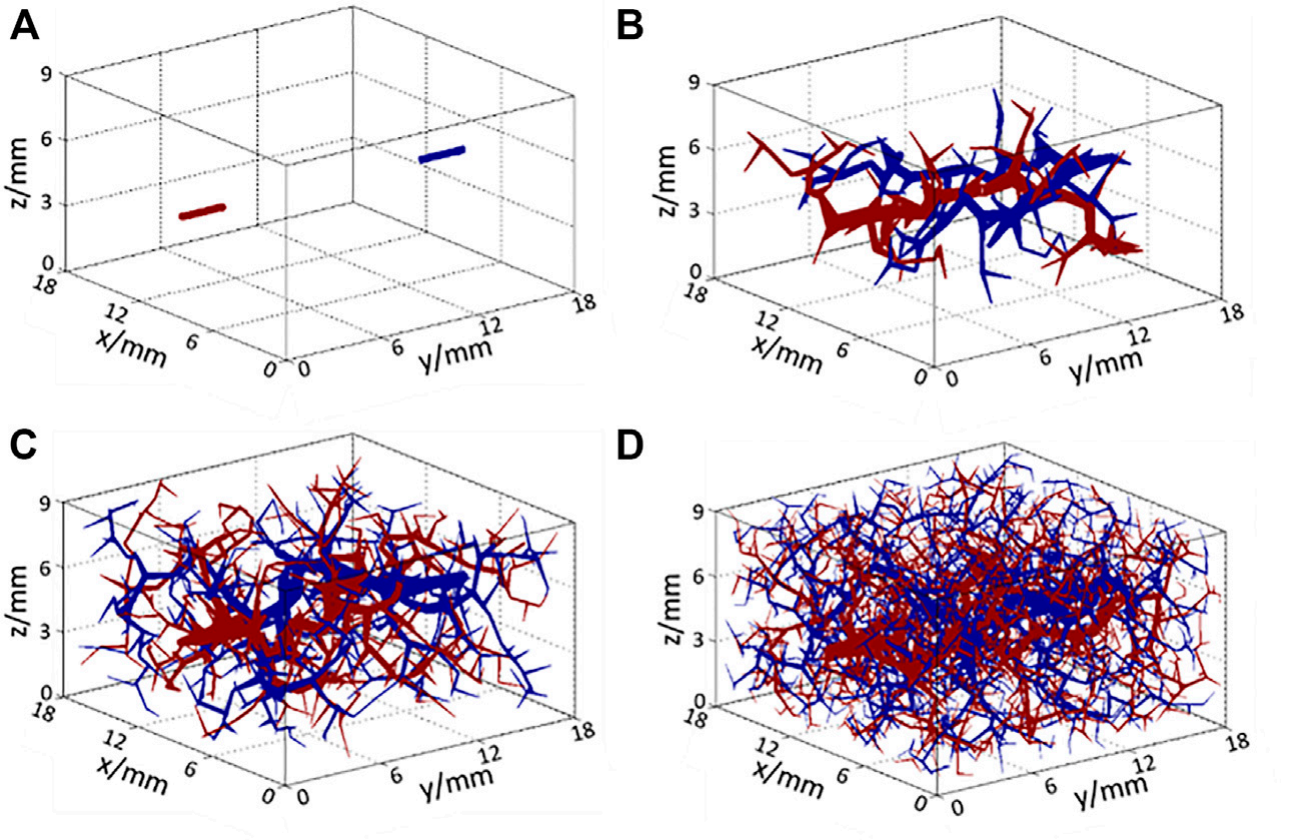
Structure of arteries (red) and veins (blue) **(A)** at the initial state and after **(B)** 100 iterations, **(C)** 500 iterations, and **(D)** 2,000 iterations of the RRT algorithm.

The subcutaneous tissue includes a large number of blood vessels to satisfy material and energy transport. As it is difficult to determine the specific structure of the blood vessels under the skin, an angiogenesis algorithm was used to generate blood vessels to fill the tissue area. In this tissue model, an inlet artery and an outlet vein were assumed inside the tissue model, as shown in Figure 3A. Then, the rapidly exploring random tree (RRT) algorithm was implemented as that in the work by Blowers et al. (Blowers, 2018) where it was used to generate brain vasculature. The procedures of the algorithm are as follows:

1) A new node is generated randomly inside the target tissue space.
2) Previous nodes are searched completely to find the closest segment or node.
3) A new segment is created by linking the created node with the closest point or with the nearest node of the nearest segment.
4) If the generated segment is connected to an existing segment (not at a node), a new node will be generated on the existing segment and will be divided in two segments.
5) The procedures 1–4 are repeated until the set number of iterations is completed.

The structures of the generated vessels after 100, 500, and 2,000 iterations of the RRT algorithm are shown in Figures 3B–D, respectively, where red lines represent arteries and arterioles, and blue lines represent veins and venules. As the number of iterations increased, blood vessels filled the entire space gradually so that blood could be perfused adequately to each part of the target tissue. In the generation of smaller vessels from the main arteries, two iteration criteria were set to end the RRT computation. If the numbers of vessels have no significant changes and the blood flow rate in the smallest vessel equals to a known value in the arteriole or venule, the computation will stop. In Figure 3D, the diameter of the smallest size of the vessel is 34 μm, which matches the size of the smallest arteriole. Additionally, to incorporate the thermal effects of capillaries, the voxel volume is regarded as a porous medium, whereby the liquid phase represents the blood in capillaries and the solid phase represents the solid tissue. Using this method, a complete multi-scale vascular and porous media model is formed. In the work, a cubic tissue and real geometric foot model have been constructed for the applications of the vascular porous media model.

#### Computation of Flow Rates in the 1D Vessel Network

Blood flow in 1D vessels is described by Poiseuille’s law. The relationship between blood velocity and pressure is described by Eq. 1:

$$u_{b}=-\frac{R^{2}}{8\mu }\frac{\partial P_{b}}{\partial s},$$ (1)

where *R* is the vessel radius, *P*_b_ is the blood pressure, ***s*** is the direction of the blood flow, *μ* is the blood viscosity, and ***u***_**b**_ is the blood velocity. The continuity equation of blood in 1D blood vessels is:

$$\pi R^{2}\nabla \cdot u_{b}=-q_{a}+q_{v},$$ (2)

where *q*_*a*_ and *q*_*v*_ denote the arterial outflow and venous inflow of blood. As blood can flow across the vessel wall and exchange with tissue at the capillary scale, *q*_*a*_ and *q*_*v*_ are set as 0 for non-terminal branches and assigned some values according to actual situations for terminal branches.

#### Computation of Flow Rates in Porous Media

In porous media, the capillary blood flow is assumed to occur *via* several thin capillaries. As such, the momentum equation can be simplified as the Darcy equation, which can be expressed as follows:

$$\nabla P=-\frac{\mu }{K}V_{\mathrm{Darcy}},$$ (3)

where *K* is the permeability of porous media, and *V*_Darcy_ is the Darcy velocity. The Darcy velocity is related to the actual velocity *V* by: *V*_Darcy_ = *εV* and *ɛ* represents the liquid volume fraction in the porous media. The continuity equation for the porous media is given as follows:

$$\rho_{b}\nabla \cdot V_{\mathrm{Darcy}}=Q_{a}-Q_{v},$$ (4)

where *Q*_a_ and *Q*_v_ are the exchanged blood flows between tissue and arteries/veins, respectively. There is no velocity perpendicular to the skin. Therefore, the boundary pressure for skin can be given as:

$${\frac{\partial P}{\partial n}|}_{skin}=0.$$ (5)

The blood flow at the interconnection between the discrete vascular and porous media are determined by the outflow of the arteries (*q*_a_) and the inflow of veins (*q*_v_) into the total ones into the tissue (*Q*_a_) and out of the tissue (*Q*_v_). The relationship between them could be written as:

$$q_{a}=Q_{a}/n;\;\mathrm{qv}=Q_{v}/m,\;$$

where *n* and *m* are the number of nodes that the peripheral arteries and peripheral veins intersect with a voxel. Outflow or inflow from the surrounding blood vessels are evenly distributed to each coupling node in this study.

#### Computation of Blood and Tissue Temperature

For simplicity, the 1D arterial vessel network, the 3D solid tissue phase, the 3D capillary phase, and the 1D venous vessel structure are denoted 1, 2, 3, and 4, respectively. The heat transfer processes in these four regions are expressed as follows:

$$V_{1}\rho_{b}c_{b}\frac{\partial T_{1}}{\partial t}=V_{1}K_{b}\left(\nabla^{2}T_{1}\right)-V_{1}\rho_{b}c_{b}U_{1}\left(\nabla T_{1}\right)+\beta_{1-2}\left(T_{2}-T_{1}\right)+\beta_{1-3}\left(T_{3}-T_{1}\right),$$ (6)

$$V_{2}\varepsilon_{2}\rho c\frac{\partial T_{2}}{\partial t}=V_{2}\varepsilon_{2}K_{c}\left(\nabla^{2}T_{2}\right)+\beta_{1-2}\left(T_{1}-T_{2}\right)+\beta_{2-3}\left(T_{3}-T_{2}\right)+\beta_{2-3}\left(T_{3}-T_{2}\right)+\beta_{2-4}\left(T_{4}-T_{2}\right)+V_{2}Q_{gen},$$ (7)

$$V_{3}\varepsilon_{3}\rho_{b}c_{b}\frac{\partial T_{3}}{\partial t}=V_{3}\varepsilon_{3}K_{b}\left(\nabla^{2}T_{3}\right)-V_{3}\rho_{b}c_{b}U_{3}\left(\nabla T_{3}\right)+\beta_{1-3}\left(T_{1}-T_{3}\right)+\beta_{2-3}\left(T_{2}-T_{3}\right)+\beta_{3-4}\left(T_{4}-T_{3}\right)+c_{b}M_{1-3}\left(T_{3}-T_{1}\right),$$ (8)

$$V_{4}\rho_{b}c_{b}\frac{\partial T_{4}}{\partial t}=V_{4}K_{b}\left(\nabla^{2}T_{4}\right)-V_{4}\rho_{b}c_{b}U_{4}\left(\nabla T_{4}\right)+\beta_{2-4}\left(T_{2}-T_{4}\right)+\beta_{3-4}\left(T_{3}-T_{4}\right)+c_{b}M_{3-4}\left(T_{4}-T_{3}\right),$$ (9)

where *V*_1–4_ denotes the volume of the domain, and *U*_1, 2, 4_ are the blood velocities in different regions. As the soft tissue is the solid phase in the porous media, *U*_3_ does not exist. *ɛ*_2_ is the volume fraction of the tissue within the porous domain, and *ɛ*_3_ is the volume fraction of the blood; therefore, *ɛ*_2_ + *ɛ*_3_ = 1. *K* and *K*_b_ are the conduction coefficients for the tissue and blood phases, respectively. *c*_b_ and *c* are the heat capacities for blood and tissue. The tissue parameters include two components which are the soft tissue and bone. *M*_1–3_ and *M*_3–4_ represent the mass exchange from domain 3 to domain 1 and from domain 3 to domain 4, reflecting convective heat transfer from arteries to voxels and from voxels to veins, respectively. *Q*_*gen*_ represents tissue metabolic heat generation. Heat exchange between the blood and the surrounding tissue through the vessel wall is described by the heat exchange coefficient *β*_*x-y*_ which is defined as follows:

$$\beta_{x-y}=\varepsilon hA_{surf},$$ (10)

where *h* is the convection coefficient of the blood and vessel wall, and *A*_surf_ is the surface area of the blood vessels. Vascular blood flow can be approximated as the laminar flow in a rigid pipe, so this convection coefficient can be deduced from the Nusselt number:

$$Nu=\frac{hD_{v}}{2K_{b}}.$$ (11)

It is well established that for a laminar flow in a pipe, *Nu* can be approximated to be a constant with the value of 4 (Blowers, 2018). At the surface of the foot model and the top surface of the cubic model, there is heat exchange with the environment and a Robin boundary condition can be prescribed as:

$$-K_{c}\frac{\partial T}{\partial n}=h\left(T-T_{\infty }\right),$$ (12)

where *T*_∞_ is the environment temperature, and *h* is the convective heat transfer coefficient. The adiabatic boundary condition was set for the plane connecting the foot to the leg in the foot model and the other surface of the cubic model:

$$-K_{c}\frac{\partial T}{\partial n}=0.$$ (13)

Having set the boundary conditions for the heat and mass transfer equations, the temperatures of the four domains could be solved with the given flow rates within domains 1, 3, and 4. A MATLAB program was developed based on an open-source code on GitHub [https://github.com/sblowers/VaPor] for the implementation of numerical computation. The physical parameters used in this computation are described in Table 2.

**TABLE 2:** Physical parameters used in the foot model.

Physical parameter	Value	Unit	References
Blood viscosity, *μ*	3.5	mPa s	Bagavathiappan et al. (2008)
Permeability of porous media, *K*	1.5 × 10^−13^	m^2^	Bagavathiappan et al. (2008)
Blood density, *ρ*_b_	1,050	kg/m^3^	Bagavathiappan et al. (2008)
Specific heat capacity of blood, *c*_b_	3,800	J/(kg K)	Bagavathiappan et al. (2008)
Thermal conductivity of blood, *K*_b_	0.50	W/m^3^	Bagavathiappan et al. (2008)
Tissue density, *ρ*_soft tissue_	1,270	kg/m^3^	Antonova et al. (2016)
Specific heat capacity of the soft tissue, *c*_soft_ _tissue_	3,768	J/(kg K)	Antonova et al. (2016)
Thermal conductivity of the soft tissue, *K*_soft tissue_	0.35	W/m^3^	Antonova et al. (2016)
Bone density, *ρ*_bone_	1,418	kg/m^3^	Antonova et al. (2016)
Specific heat capacity of the bone, *c*_bone_	2,409	J/(kg K)	Antonova et al. (2016)
Thermal conductivity of the bone, *K*_bone_	2.21	W/m^3^	Antonova et al. (2016)
Metabolism, *Q*_gen_	368	W/m^3^	Antonova et al. (2016)

In this model, ***u***_***b***_ represents the velocities in blood vessels including the velocities in arteries (***U***_**1**_) and veins (***U***_**4**_) which are solved by Eqs 1 and 2. The vessel network model used in this work is a 1-dimensional one with the information of radii and lengths. In the computation of the blood flow of the 1D model, the velocity in every segment is computed by Eq. 1, while the velocities at the bifurcation nodes should be computed by combining Eqs 1, 2 to satisfy the continuity condition of mass flow at these points. The direction of the blood flow in every segment depends on the known position of every vessel segment generated by the RRT algorithm. The direction of vector ***u***_***b***_ refers to the one in the 3D information of the network. Similar implementation can be found in the works of Pozrikidis (Pozrikidis, 2009) and Blowers, (2018) for blood flow simulation through vascular networks. On the other hand, ***U***_**3**_ can be obtained by solving Eqs 3, 4. ***V***_**Darcy**_ represents the Darcy velocity in the porous medium model which is related to the velocity of the blood flow in a capillary (***U***_**3**_).

Since voxel-based meshes are employed, the finite difference method (FDM) can be easily employed for the discretization of Eqs 6–9. The real geometric 3D model reconstructed from medical images can also be directly transformed into a voxel mesh for the computation by the FDM. Currently, the steady-state temperature is considered; thus, the discretization of the time derivative term is not needed. The convection term is discretized using the first-order upwind difference scheme. The diffusion terms of the equations are discretized using the central difference scheme. The discrete governing equation can be then written as a stiffness-matrix form as:

Ax=B, (14)

where ***x*** is the temperature matrix, *A* is the coefficient matrix of temperature, and *B* is the loading term derived from the known terms in the governing equations. Through the built-in Gaussian elimination algorithm in MATLAB, the above algebraic equation can be solved. It takes about 10 min in MATLAB on a PC with an Intel i7 6700k QuadCore processor and 32 GB of RAM for a foot model with a 1.5 mm-voxel size and 50,000 generated vessels.

## Results

The process of the thermal analysis on the cubic tissue and foot model are basically the same, among which the heating source should be taken into account for the cubic model. Since the measurement period is sufficiently long, the stable heating and power-off instance were chosen for the analysis in the cubic tissue model. The blood temperature of the inlet artery is constant at 37°C. The convective and radiative boundary condition is assigned at the skin of which the total heat transfer coefficient was set to be 8.0 W/m^2^ K. The environmental temperature was set as 23°C, following the same environmental condition of Sivanandam et al. (2012). The results of the heat transfer computation in the two models are presented in *Tissue Temperature Distributions Under Heating and Power-Off Condition and Temperature Distributions for Different Vasculatures on Foot* sections, respectively. The inversion of blood flow for healthy people and diabetic patients is illustrated in *Inverse Analysis of Skin Blood Flow in Healthy People and Diabetic Patients* section.

### Tissue Temperature Distributions Under Heating and Power-Off Condition

**FIGURE 4:**
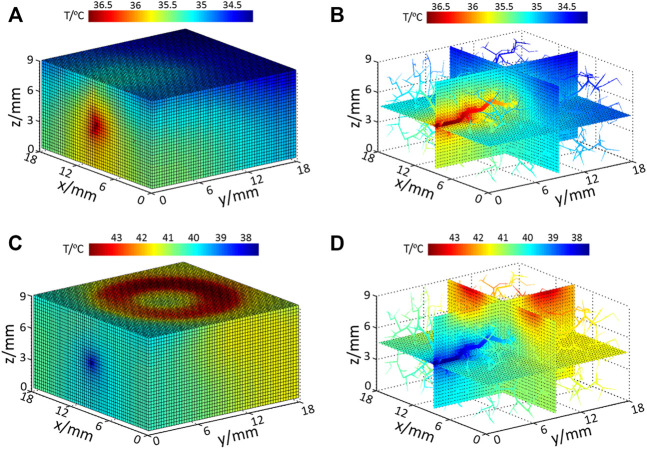
Temperature distribution of **(A)** model surface with no input heating power; **(B)** internal tissue and vessel temperature distribution with no extra heating power; **(C)** model surface temperature under heating and **(D)** internal tissue and vessel temperature under heating.

When there is no external heat stimulation on the skin (resting and recovery stage), the computed tissue temperature distribution is shown in Figure 4A, and the temperature distribution of internal tissue sections coupling with vessels is displayed in Figure 4B. In this phase, blood flow is the heat source; thus the highest temperature is located at the arterial entrance. As illustrated in Figure 4B, the temperatures of blood vessels gradually decrease along the flow direction due to the heat exchange between the blood vessel and the surrounding tissue. At the skin surface, it is seen that the skin temperature in the upstream is slightly higher than that in the downstream position.

When the skin surface is heated by the SPAS (heating stage), the temperature of the heated ring zone at the skin surface increases significantly, as shown in Figure 4C. At this time, blood plays a role of heat dissipation; thus, the coolest area is located at the arterial inlet. Figure 4D shows the temperature distribution of the inner section and blood vessels. As the blood is heated during flowing, the temperature of blood at the downstream is increasing; thus, the cooling effect of the blood flow in the downstream is weaker than that in the upstream of the blood flow. This effect is also reflected in the skin surface that the surface skin temperature in the downstream area of the heating ring is higher than that in other areas.

### Temperature Distributions for Different Vasculatures on the Foot

**FIGURE 5:**
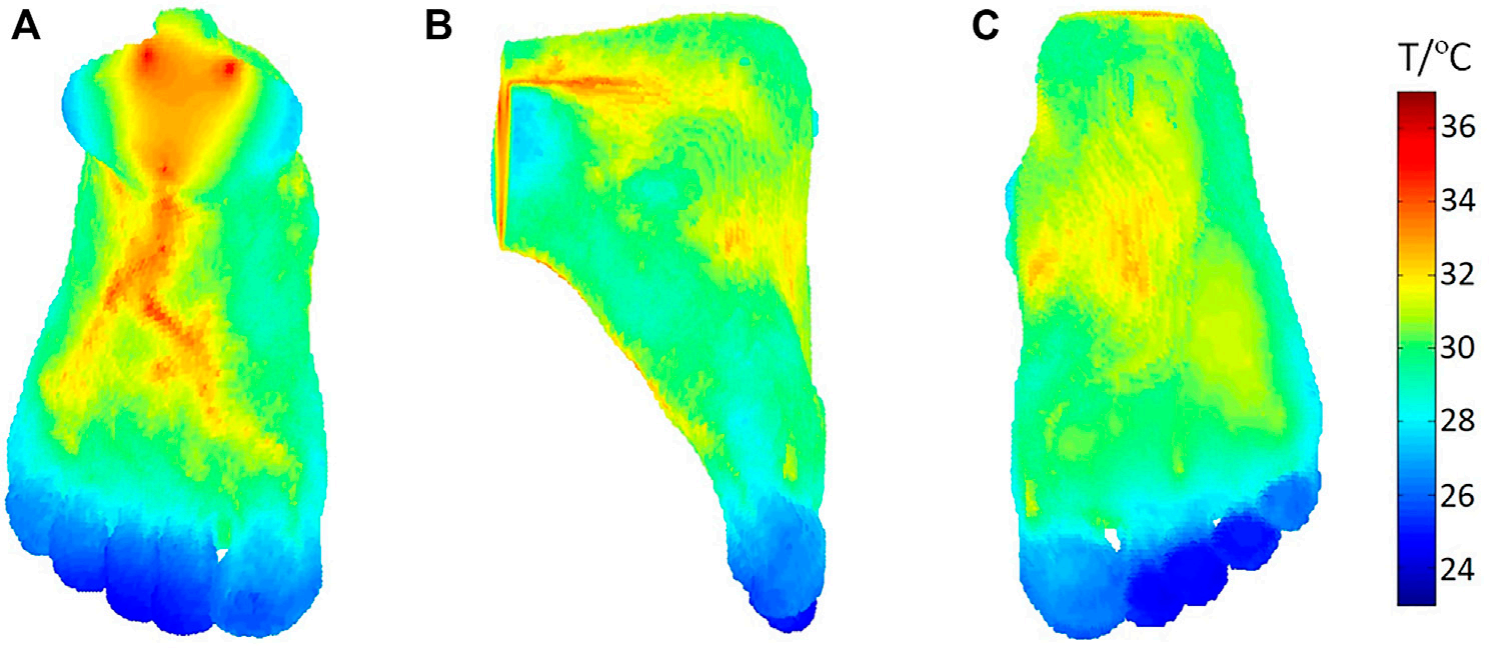
Temperature distribution of a foot from **(A)** dorsal, **(B)** side, and **(C)** plantar view.

The vascular—porous media model has been applied in the thermal analysis on the foot and for establishing a quantitative relationship between the blood flow and temperature distribution. The foot model in this study was reconstructed from sequential medical images. Simpleware software (Exeter, United Kingdom) was used to identify the bone and soft tissue automatically from CT images. The structure of the basic blood vessel of this foot model was obtained from the available website (https://human.biodigital.com). and further developed by using the RRT algorithm. Figure 5 shows the foot skin temperature distribution from a dorsal, side, and plantar view. The temperature ranges from 24.28°C to 32.12°C. In Figure 5A, it can be observed that the temperature of the skin near the large blood vessels is higher, and it decreases gradually to the distal end. In Figure 5C, the computed average temperature of the foot plantar region is 29.42°C, which is 0.5°C higher than the experimental data (Sivanandam et al., 2012). In addition, the computed temperature distribution shows the temperature of the arch is higher than that of the sole and heel, and the toes are the coldest area of the whole foot. Specifically, the 1^st^ and 5^th^ toes are warmer than the 2^nd^, 3^rd^, and 4^th^ toes. The computed temperature values closely approach the experimental data, and the abovementioned characteristics for the temperature distribution are consistent with the standard thermographic patterns of feet (Alfred et al., 2015), confirming the validity of the vascular porous media model.

**FIGURE 6:**
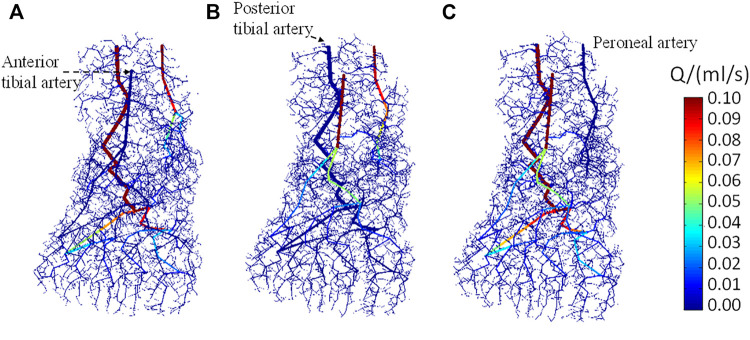
Dorsal view of the blood flow in arteries when stenosis occurs in **(A)** anterior tibial artery, **(B)** posterior tibial artery, and **(C)** peroneal artery, respectively.

Figures 6A–C show the distribution of arterial blood flow when occlusion occurs in the anterior tibial artery, posterior tibial artery, and peroneal artery, respectively. The occlusion positions in the three inlet arteries were specifically marked, wherein the anterior tibial artery supplies blood to the dorsal part of the foot, the posterior tibial artery supplies blood to the plantar region, and the peroneal artery supplies blood to the lateral areas. The vasculature in the foot model can be also seen clearly. As the total number of vessels is more than 1 × 10^6^, it is difficult to visualize all vessels concurrently; only the vessels with diameter >100 μm are displayed. It is observed that the blood flow in the non-blocked inlet vessels and its downstream vessels gradually decreases along with the bifurcation generations. Apart from the blood flow in the major blood vessels, the arteriole and venule flow rates are <0.01 ml/s, indicating that the blood had been perfused to all parts of the foot. In contrast, no blood flows through the downstream area of the blocked vessel which will affect the distribution of the foot temperature.

**FIGURE 7:**
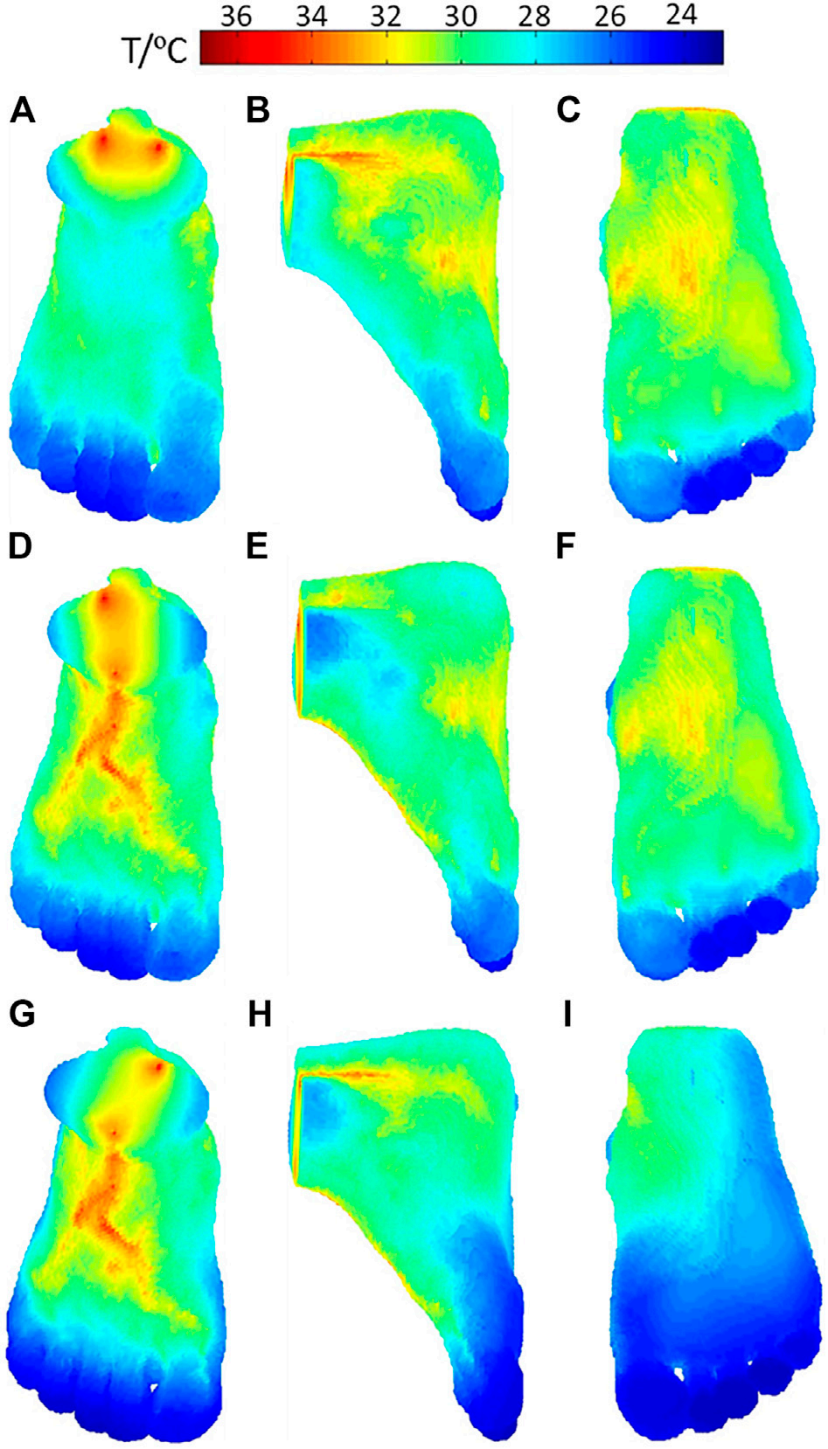
Temperature distribution of the foot model when stenosis occurs in **(A–C)** anterior tibial artery occlusion, **(D–F)** posterior tibial artery, and **(G–I)** peroneal artery.

The temperature distributions on the surface of the foot when the three inlet vessels are blocked, as shown in Figure 7, where the rows indexed (a–c), (d–f), and (g–i) correspond to the temperature contours for the occlusion of the anterior tibial artery, posterior tibial artery, and peroneal artery, respectively. When the anterior tibial artery is occluded, the temperature of the upper surface of the foot decreases considerably, especially in the central region more influenced by the occluded feeding artery. If observed from the lateral side and foot sole, as shown in Figures 7B,C, there are no remarkable changes in the temperature compared to the healthy case. As the blood flow in the peroneal artery is at the normal state, the temperature decreases in the heel are small. This means when occlusion occurs in the anterior tibial artery, the most affected area in the skin temperature is the plantar surface. Moreover, in the middle and lower part of the foot, especially the toes, the lower temperature areas are enlarged which can even be seen from the dorsal and side view. Similarly, as seen in Figures 7D–I, the occlusions of the posterior tibial and peroneal artery have the impacts on the sole and lateral side of the foot. Meanwhile, it is noted that the occlusion in the posterior tibial artery seems to result in the largest lower temperature area, suggesting that the occlusion in the posterior tibial artery should be paid more attention to.

### Inverse Analysis of the Skin Blood Flow in Healthy People and Diabetic Patients

**FIGURE 8:**
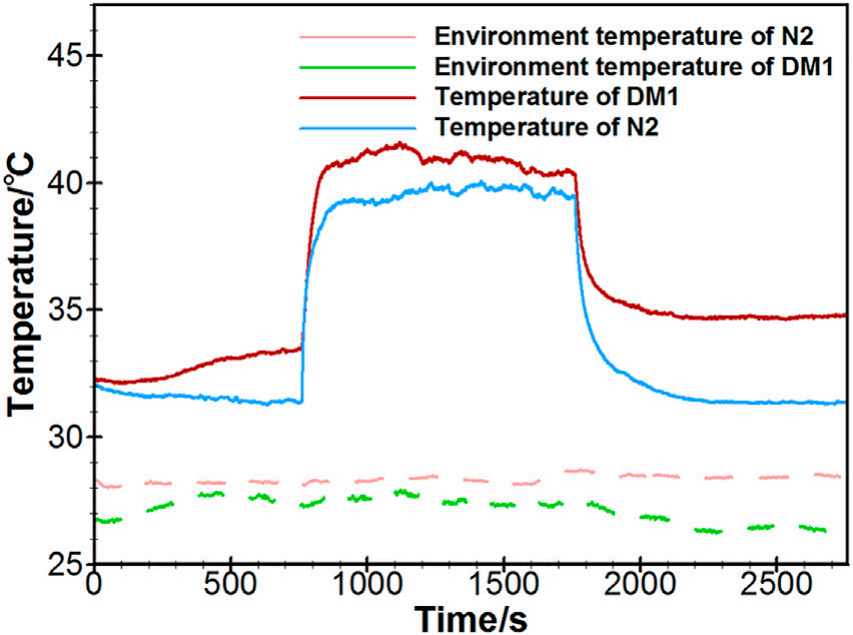
Measured temperature curves by the SPAS for a healthy person and a diabetic patient.

The vascular porous media model has applied in the thermal analysis of blood flow regulation in the SPAS test. The measurements by using the SPAS were implemented on five healthy people denoted by N1∼N5 and 31 diabetic patients denoted by DM1∼DM31. The experiments have been approved by the Biological and Medical Ethics Committee of Dalian University of Technology. The data collection of diabetic patients was performed in the First Affiliated Hospital of Anhui Medical University. The ages, BMI indices, and blood glucose levels of the subjects are shown in Table A1 as an Appendix. The temperature curves of a typical diabetic patient (DM1) and healthy people (N2) are shown in Figure 8. In each phase, the temperature of the diabetic patient is higher than that of the healthy person. In the heating phase, both the temperature rises rapidly and then stabilizes for a sufficiently long period. However, after heating is over, the temperature of the healthy person returns to the level before heating, but the temperature of the diabetic patient has not declined to the resting stage range.

**FIGURE 9:**
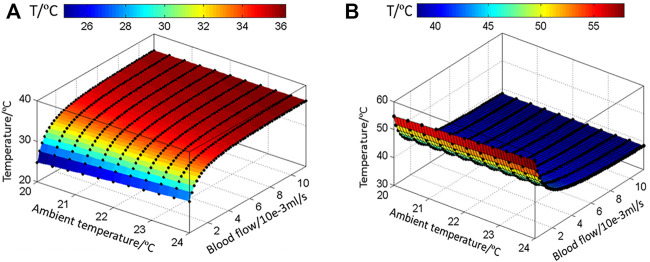
Fitted curves to express the relationships of the blood flow, ambient temperature, and skin temperature in the **(A)** recovery and **(B)** heating phase.

The ambient temperature varies slightly in different tests, as shown in Figure 8, since the tests were carried out during a short period of several days. In order to estimate the blood flow in each test, the temperature distributions of the cubic tissue model were computed for various inlet flow rates from 0 to 0.01 ml/s, and the ambient temperature was set to 20 to 24°C, respectively. The skin temperature at the center point surrounded by the heating ring was subsequently extracted. A set of curves for the relationships between the skin temperature, blood flow, and ambient temperature were achieved and could be fitted as a two-dimensional graph by using the surface fitting tool toolbox in MATLAB. Figures 9A,B give the temperature variations at the recovery and heating phase. It is clear to see that the skin temperature increases with the input blood flow rate without external heating; meanwhile, the increase of the environmental temperature can also lead to the slight increase of the skin temperature. If an external constant heat is added, the center-point skin temperature decreases with the input blood flow rate. Temperature varies significantly when the input blood flow changes from 0 to 0.002 ml/s and then tends to vary slowly when the blood flow is further increased. Having the set of fitted curves, the input arterial blood flow rate could be determined from the measured environmental and skin temperatures.

**FIGURE 10:**
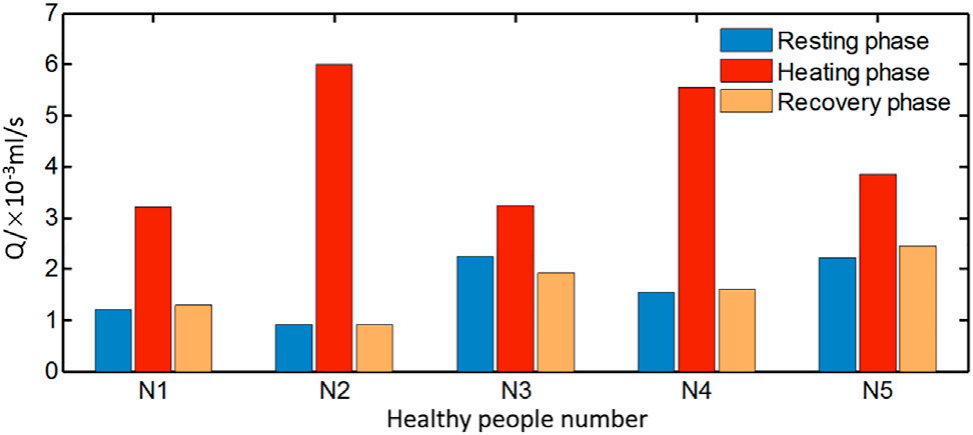
Average blood flow rate of healthy subjects during rest, heating, and recovery phases.

In order to analyze the blood flow during the stable period of each stage, the measured temperature data of the last 100 s in each stage were extracted to evaluate the average blood flow during resting, heating, and recovery stages. The obtained results for the healthy people are displayed in Figure 10. The typical blood flow variation pattern for the healthy subjects is the blood flow rates at the resting and recovery phases, which are distinctly lower than that at the heating stage although there are slight differences among the healthy subjects. The blood flow increases at the heating stage since the blood vessels dilate for heat dissipation when the skin is heated. The smallest blood flow at the heating phase is 0.32 ml/s which is larger than the largest blood flow rate of 0.23 ml/s at the resting phase among healthy people. At the recovery phase after the power is off, the blood flow returns to the resting level before heating with the difference of less than 10%.

**FIGURE 11:**
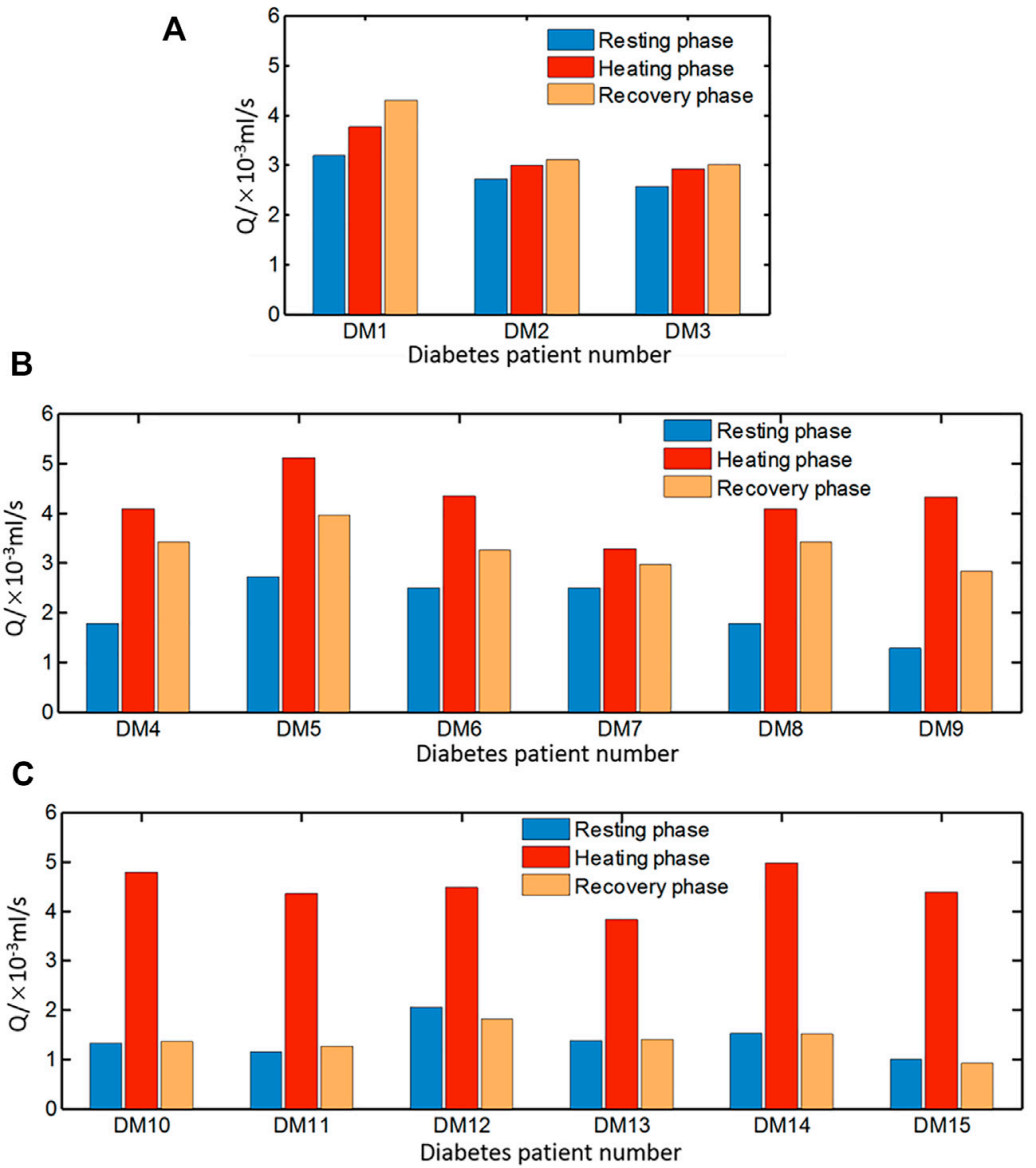
Average blood flow rate of diabetic patients in three groups **(A)** DM1–DM3, **(B)** DM4–DM9, and **(C)** DM10–DM15 during resting, heating, and recovery phases.

However, the blood flow variation patterns of individuals with diabetes vary greatly, especially at the recovery stage. According to the blood flow alteration patterns at the resting and recovery stage, the estimated results can be classified into three groups shown in Figures 11A–C, respectively. In Figure 11A, the blood flow rate during heating is greater than that of the resting phase, but the increment is smaller compared to that of healthy people. It is noticeable that the blood flow rate at the recovery stage is even larger than that at the heating stage. In Figure 11B, the variation pattern of the blood flow rate at the resting and heating stage is close to that of healthy subjects; however, the blood flow rate at the recovery stage is only slightly reduced from the value at the heating stage, whose value is close to the average one of the resting and heating stage. Seven sets of data in the third group are shown in Figure 11C. The pattern of the blood flow variation for the group of diabetic patients is similar to that of healthy subjects that the blood flow rate at the resting and recovery stage are remarkably lower than the one at the heating stage.

## Discussion

In this study, thermal analysis of blood flow was performed in a cubic tissue and a real geometric foot model by using a discrete vascular-porous media model. The discrete vascular-porous media model was first put forward by Blowers et al. in analyzing the effect of therapeutic hypothermia of the human brain. It is our opinion that the advantage of the model is to take into account the influence of the vasculature more precisely. Hence, a MATLAB program was further extended to allow the model to calculate more complex conditions with a ring-mounted vessel structure and a vascular stenosis. The blood flow rates in vasculatures and temperature distributions have been achieved under various thermal conditions, providing the possibilities to analyze blood flow conditions in the tissue from the surface temperature.

In analyzing the thermal responses measured by the SPAS, the inlet arterial blood flow of the three stages was estimated and compared between healthy subjects and diabetic patients. It is seen that the blood flow rate at the recovery phase fell back to the level at the resting phase for healthy subjects. However, in the diabetic group, the blood flow in the recovery phase is close to the blood flow rate at the heating phase and even larger than it, which manifests that the vasodilation under a thermal stimulation is delayed in some of the diabetic patients. The results further support our previous studies (Wang et al., 2020) on SD rats that the blood flow drops rapidly after thermal stimulation for all healthy rats, but the delay of the declination occurs in some diabetic rats. Compared to the 2nd blood flow variation pattern in diabetic patients, the 1st blood flow variation pattern suggests more delayed time to thermal response. It is implied that the diabetes with delayed autoregulated vasomotion may be suffering from peripheral vascular diseases and needs to be more concerned. By comparing the analytical results to the clinical diagnoses by the First Affiliated Hospital of Anhui Medical University for the 15 diabetic subjects, it is found that except one patient, the other patients are suffering different kinds of complications, including hypertension, peripheral vascular disease, or peripheral neuropathy.

Both the skin temperature variation in diabetic patients and rats in our experiments show the similar pattern that the severe the diabetic mellitus is, the faster the skin temperature increases. Although constant thermal conductivity and the same vasculature were assigned in the work, it will not affect the estimated blood flow pattern in healthy and diabetic patients. Nonetheless, the possibility still exists that some estimated healthy blood flow patterns may have some abnormalities due to neglecting the impacts of aging and fat thickness.

Aging may cause the decrease of the microvasculature density and increase of vessel stiffness, leading to the increase of peripheral resistance. This kind of alteration can be also reflected in the change of effective conductivity. If microvasculature density decreases, the effective thermal resistance will increase. Similarly, fat thickness increasing will increase the thermal resistance as well. These factors can lead to faster temperature increasing in the heating stage of the test and reinforce the predicted blood flow variation pattern, which is with a higher ratio of the blood flow in the recovery phase to that in the heating phase. In addition, the increase of thermal resistance can result in a damping of the magnitude of the skin temperature oscillation and a larger phase lag to the blood flow (Tang et al., 2017).

It is commonly known that the foot temperature of diabetic patients is lower which is caused by the lower blood perfusion rate (Uraiwan et al., 2018). Equations 6–9 have the similar form of the Pennes equation, but they describe the heat transfer process of arteries, tissue, capillaries, and veins more in detail rather than using a lumped source/sink term in the Pennes equation. The vascular-porous media model captures the thermal effect of heat conduction and convection within their regions and between blood vessels and their surrounding tissues. The alterations of blood vessel structures could also be explored. The Pennes equation could be applied in thermal analysis of foot, but it needs more assumptions for blood perfusion. The computed temperature of the foot *via* the vascular-porous media model is in a favorable agreement with thermography, showing the prospective applications in the assessment of vascular alterations for a diabetic foot.

With the development of medical imaging technology such as laser speckle flowmetry or thermography, it has been possible to directly obtain structural lesions of the blood vessels of body parts. However, early detection of the diabetic foot needs to be performed at a high frequency. Medical imaging testing is not yet suitable for daily inspections due to the high cost. Thermography is a prospective way for screening the microvasculature, but a more reliable algorithm for converting the temperature to blood flow is needed. The voxel-based vascular-porous media model provides a useful way for the conversion. Additionally, the medical image could only reflect the structural lesions but not functional lesions. It is found that the diabetic vascular functional lesions may appear before structural lesions through rat experiments (Wei et al., 2021). Coupling with thermography and the porous media model, the stenosis degree of peripheral vessels, vascular density variation, or vasodilation dysfunction may be detected, which are meaningful for the early diagnosis of a diabetic foot.

In our previous study, we measured the foot and finger temperature simultaneously and found that the foot skin temperature is more sensitive to reflect the endothelial dysfunction, but finger temperature can also give the same information (Tang, 2017). Since vascular abnormalities frequently occur in a similar fashion (Abularrage et al., 2005), abnormal vasomotion of hand could reflect the foot vascular disease to a certain extent. Compared to foot, hand is more accessible for measurement. Despite that, we did not focus on the coherence between the data in the hand and foot; there are three patients suffering peripheral neuropathy among the screened abnormal blood flow patients through the thermal analysis of SPAS data. This reveals that the hand skin temperature can also be a tracer for the early detection of the diabetic foot. More data from hand and foot should be collected simultaneously and compared for showing the coherence between them in detecting the endothelial dysfunction.

Additionally, although the relationship between the temperature and blood flow distribution was established in the foot model, the inversion of the blood flow of inlet arteries of the foot is not as simple as the process in the cubic model. Different inlet blood vessels or intermediated blood vessels own their respective temperature influencing areas, and the abnormal temperature distribution is a combined result of multiple blood flow under different degrees of stenosis. Therefore, in the future, it is necessary to divide the foot into several areas and apply available optimization methods to comprehensively investigate the coupling effect of various vascular disorders on temperature.

In generation of the foot vasculature, the nodes of the main blood vessels of the foot were manually set with reference to the physiological model obtained from the website, where the distance between the vessel and bone can be determined from the slices of the foot. After setting the arterial and venous nodes, the vessel network was compared to the physiological model again. Although the website provides the detailed structure of the foot including all tissues, the specific data could not be obtained. Thus, we manually made a vessel network according to the relative distance and size of the physiological model and inserted into the voxel-based model. The names and positions of the foot blood vessels in our model are also in agreement with the ones in the literature (Manzi et al., 2011).

**FIGURE 12:**
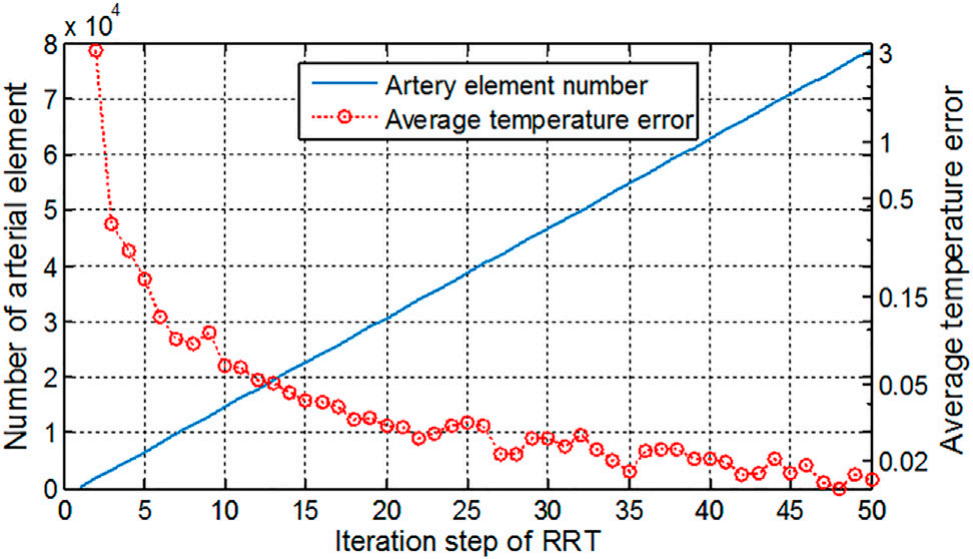
Artery element number and average temperature error along with the increment of iteration steps in the RRT algorithm.

The RRT algorithm was implemented to generate the blood vessels so that the blood is perfused into various regions of the tissue. Figure 12 shows the number of vessel elements and relative temperature errors along with the increasing RRT iterations. The number of iteration steps increases by 1,000 step each time, and the mean square error of the surface temperature between the newly computed temperature and original one was computed. The initial average error reached 3°C and gradually reduced as the iteration number increases. It is seen that the more iteration steps are used, the smaller blood vessels can be included and larger computational burden is resulted in. At the 50,000 s′ step, the average temperature error has been reduced to 0.01°C, and temperature distribution has hardly changed even with more iteration steps. Therefore, the iteration of this foot model is set as 50,000. In the computation for the cubic tissue model, the iteration step is set as 2,000 due to the smaller model size.

Despite of the reasonable structure and sizes of the vessels, it should be noted that the model has not reflected the individual differences in the foot structure. Our rat experimental study has shown that the microvasculature alteration occurs after the endothelial dysfunction (Wei et al., 2021). In the current study, it is assumed that the microvasculature has not been changed since the blood flow alterations due to the change of vasodilation is the first aim of the work. On the other hand, the iteration number of the RRT algorithm may give an impact on the structure of the vascular network and further influence the blood flow in a one-dimensional straight tube model. In the future we may optimize the RRT algorithm in the setting of the bifurcation angle of blood vessels and fractional dimensions so that various altered vasculatures in pathological conditions can be generated. Using MR angiography for showing the main vessels is an alternative way as well.

## Conclusion

In this study, a vascular-porous media model has been applied in the thermal analysis of foot and a cubic tissue. Since the blood flow and the heat exchange within tissues during the flow process was considered by using a multiscale model, the temperature distributions under various thermal and blood flow conditions can be achieved so that the non-linear relationship between the blood flow and skin temperature can be further deduced. The computed results in the foot model reveals that the stenosis of feeding arteries can lead to temperature decreases in the downstream for different degrees, among which the occlusion of the posterior tibial artery has the largest lower-temperature area.

Then, analysis of the thermal response test imposed on type 2 diabetic patients and healthy subjects show that for a healthy subject, the blood flow rate after heating power is off at the recovery stage decreases to the level as that of the resting condition, whereas in some diabetic patients, the blood flow rates solely decrease slightly or even increase further at this stage. This implies that the vasodilation function to the thermal stimulus is delayed in the subject, which verifies the conclusions in our previous studies on diabetic rats. Most of the screened diabetic patients with peripheral vascular or neuropath disease are in agreement with those diagnosed clinically. It is believed that the discrete vascular-porous media model may have more applications, especially to studies of the diabetic foot by coupling with thermography imaging.

## Data Availability

The raw data supporting the conclusions of this article will be made available by the authors, without undue reservation.

## Appendix

**TABLE A1:** Information on healthy people and diabetic patients in the experiments.

Number of subject	Age	BMI
N1	52	22.0
N2	23	20.55
N3	23	18.21
N4	25	25.09
N5	28	28.34
DM1	35	20.99
DM2	48	33.14
DM3	44	25.0
DM4	32	25.69
DM5	57	28.88
DM6	64	28.7
DM7	47	26.85
DM8	49	29.39
DM9	50	30.0
DM10	34	28.53
DM11	37	32.57
DM12	50	26.9
DM13	51	22.14
DM14	66	27.9
DM15	57	25.25
